# Function and cancer genomics of *FAT* family genes

**DOI:** 10.3892/ijo.2012.1669

**Published:** 2012-10-17

**Authors:** MASARU KATOH

**Affiliations:** Division of Integrative Omics and Bioinformatics, National Cancer Center, Tokyo 104-0045, Japan

**Keywords:** FAT, YAP, TAZ, actin dynamics, G-protein-coupled receptor, receptor tyrosine kinase, WNT, β-catenin, cancer stem cells, whole-genome sequence

## Abstract

FAT1, FAT2, FAT3 and FAT4 are human homologs of *Drosophila* Fat, which is involved in tumor suppression and planar cell polarity (PCP). FAT1 and FAT4 undergo the first proteolytic cleavage by Furin and are predicted to undergo the second cleavage by γ-secretase to release intracellular domain (ICD). Ena/VAPS-binding to FAT1 induces actin polymerization at lamellipodia and filopodia to promote cell migration, while Scribble-binding to FAT1 induces phosphorylation and functional inhibition of YAP1 to suppress cell growth. *FAT1* is repressed in oral cancer owing to homozygous deletion or epigenetic silencing and is preferentially downregulated in invasive breast cancer. On the other hand, *FAT1* is upregulated in leukemia and prognosis of preB-ALL patients with *FAT1* upregulation is poor. FAT4 directly interacts with MPDZ/MUPP1 to recruit membrane-associated guanylate kinase MPP5/PALS1. FAT4 is involved in the maintenance of PCP and inhibition of cell proliferation. *FAT4* mRNA is repressed in breast cancer and lung cancer due to promoter hypermethylation. *FAT4* gene is recurrently mutated in several types of human cancers, such as melanoma, pancreatic cancer, gastric cancer and hepatocellular carcinoma. FAT1 and FAT4 suppress tumor growth via activation of Hippo signaling, whereas FAT1 promotes tumor migration via induction of actin polymerization. *FAT1* is tumor suppressive or oncogenic in a context-dependent manner, while *FAT4* is tumor suppressive. Copy number aberration, translocation and point mutation of *FAT1, FAT2, FAT3, FAT4, FRMD1, FRMD6, NF2, WWC1, WWC2, SAV1, STK3, STK4, MOB1A, MOB1B, LATS1, LATS2, YAP1* and *WWTR1/TAZ* genes should be comprehensively investigated in various types of human cancers to elucidate the mutation landscape of the FAT-Hippo signaling cascades. Because YAP1 and WWTR1 are located at the crossroads of adhesion, GPCR, RTK and stem-cell signaling network, cancer genomics of the FAT signaling cascades could be applied for diagnostics, prognostics and therapeutics in the era of personalized medicine.

## Contents

1. Introduction
2. FAT family
3. Processing of FAT proteins
4. Signaling and function of FAT1 and FAT4
5. Cancer genomics of *FAT* family genes
6. Conclusion
7. Perspectives

## 1. Introduction

**Figure 1:**
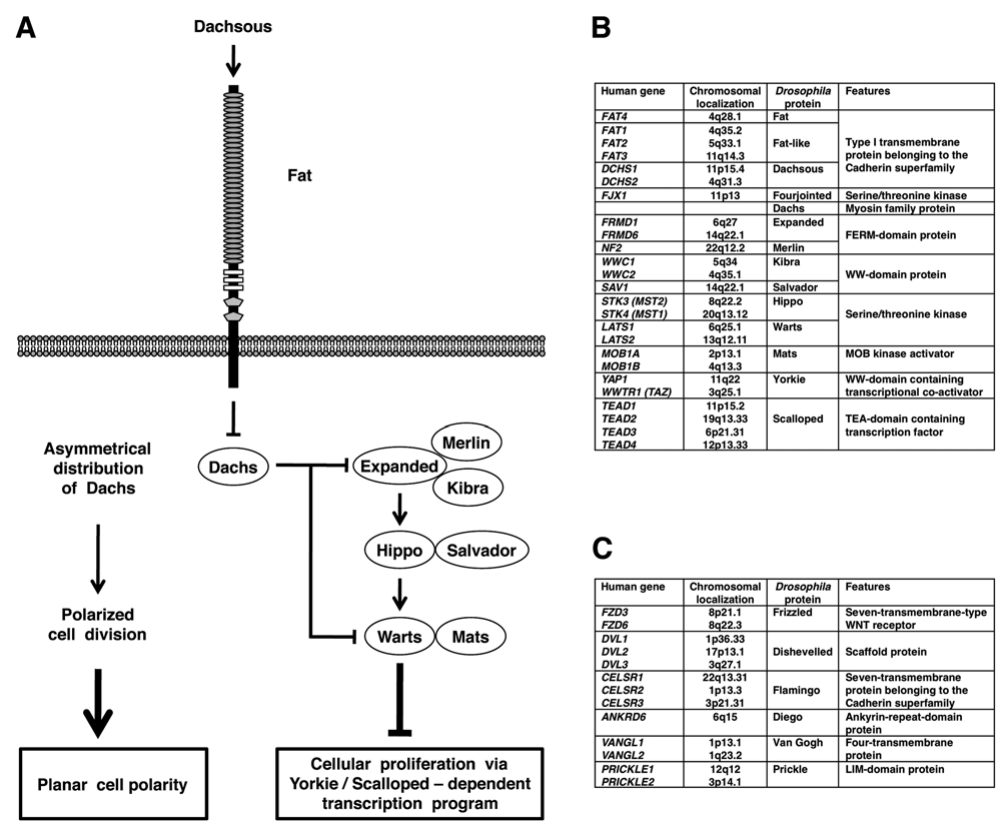
*Drosophila* and human Fat signaling components. (A) *Drosophila* Fat signaling cascades. *Drosophila* Fat is involved in the Hippo as well as planar cell polarity (PCP) signaling cascades. (B) Human orthologs of *Drosophila* genes involved in the Fat-Hippo signaling. (C) Human orthologs of *Drosophila* genes involved in the core PCP signaling.

*Drosophila* mutants of the *fat*, *discs large* (*dlg*), *lethal giant larvae* (*lgl*), *warts*, *scribble, salvador* and *hippo* genes show tissue overgrowth (1–7). Overgrowth mutants of *fat*, *warts*, *salvador* and *hippo* are characterized by hyperplastic tumors mostly retaining single-layered epithelial structure, whereas those of *scribble, dlg* and *lgl* are characterized by neoplastic tumors losing epithelial structure (8,9). *Drosophila fat* gene is genetically upstream of the *warts*, *salvador* and *hippo* genes, which are involved in the repression of Yokie-Scalloped-dependent transcription of *cyclin E* and *diap1* genes (10–13). Because *cyclin E* and *diap1* genes encode cell cycle accelerator and apoptosis inhibitor, respectively, loss-of-function mutations of *Drosophila fat* gene give rise to hyperplastic tumors through increased cell proliferation and decreased cell death (Fig. 1A).

In addition to tumor suppression, *Drosophila fat* gene is involved in planar call polarity (PCP) (Fig. 1A). PCP is the cell polarity within the plane of epithelial tissues orthogonal to the apical-basal axis (14–17). PCP is established as a result of the asymmetrical localization of the Flamingo-Frizzled-Dishevelled-Diego complex and the Flamingo-Strabismus-Prickle complex of adjacent cells via homophilic interaction of extracellular cadherin-repeat region of Flamingo. *Drosophila frizzled, dishevelled, diego, flamingo (starry night), strabismus (van Gogh)* and *prickle* genes encode the core PCP components (18–20), while *Drosophila fat, dachsous, four jointed*, *discs overgrown* and *dachs* genes encode the additional or complementary PCP components (21–24).

*Drosophila fat* gene encodes a large transmembrane protein with 34 Cadherin repeats, 4 EGF-like domains and 2 Laminin G-like domains in the extracellular region (25). Fat protein belongs to the Cadherin superfamily, which is classified into the classical cadherin family, Flamingo/Celsr family, Fat/Dachsous family and others (26,27). Extracellular regions of Fat and Dachsous cadherins on adjacent cells are reported to preferentially interact in a heterophilic manner (14,15). Four jointed and Discs overgrown are serine/threonine kinases that phosphorylate extracellular domain of Fat in the Golgi and intracellular domain of Fat in the cytoplasm, respectively, to promote Fat signaling (21–24). Heterophilic interaction of Fat and Dachsous cadherins leads to asymmetrical localization of Dachs myosin; depletion of Dachs in the Fat side and accumulation of Dachs in the Dachsous side. Asymmetrical Dachs localization induces PCP through tension anisotrophy-oriented cell rearrangement as well as tumor suppression though Hippo-Salvador-Warts signaling-mediated Yorkie repression (Fig. 1A).

*Drosophila* components of Fat-Hippo and Fat-PCP signaling cascades are well conserved in mammals, especially in human (Fig. 1B and C). Although precise mechanisms of the Fat-Hippo and Fat-PCP signaling cascades are not completely elucidated, growing pieces of evidence indicate the involvement of the mammalian FAT signaling cascades in embryogenesis and carcinogenesis. In this report, function and cancer genomics of the human FAT family members are reviewed.

## 2. FAT family

The human *FAT* gene family consists of the *FAT1*, *FAT2*, *FAT3* and *FAT4* genes (28–31). Dunne *et al* reported complete coding sequence of *FAT1* in 1995. Wu and Maniatis reported complete coding sequence of *FAT2* in 2000. Höng *et al* reported partial coding sequence of *FAT3* in 2004. We reported complete coding sequence of *FAT3* and *FAT4* in 2006. The *FAT1* and *FAT3* genes adjoin the *MTNR1A* and *MTNR1B* genes, respectively. *FAT1* is most homologous to *FAT3*, while *MTNR1A* is most homologous to *MTNR1B*. These facts clearly indicate that the *FAT1*-*MTNR1A* locus on human chromosome 4q35.2 and the *FAT3*-*MTNR1B* locus on human chromosome 11q14.3 are paralogous regions within the human genome (31).

**Figure 2:**
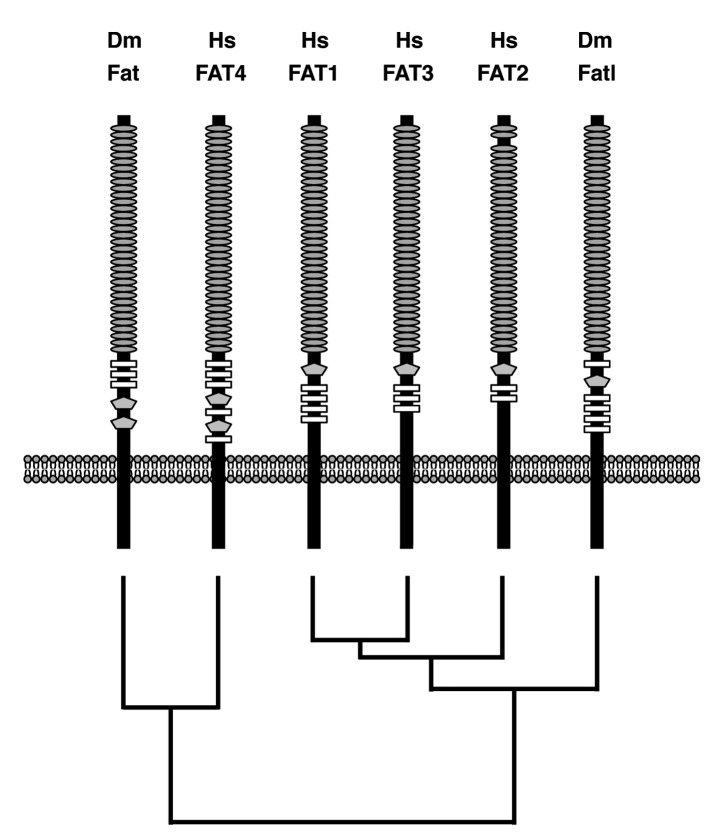
Domain architectures and phylogenetic tree of human and *Drosophila* Fat family members. Hs, human; Dm, *Drosophila*; grey oval, Cadherin repeat; open rectangle, EGF-like domain; gray pentagon, Laminin G-like domain. Human FAT1, FAT2 and FAT3 are orthologs of *Drosophila* Fat-like (Fatl), whereas human FAT4 is the ortholog of *Drosophila* Fat.

Human *FAT* family genes as well as *Drosophila* Fat family genes encode large proteins with extracellular Cadherin repeats, EGF-like domains, and Laminin G-like domain(s). Codon 275–352 of FAT2 is homologous to the third Cadherin repeat of FAT1; however, this region of FAT2 was not predicted as the Cadherin repeat using the conserved domain search (CDS) program of NCBI. Codon 3790–3828 of FAT1 and codon 3799–3834 of FAT3 are distantly related to the EGF-like domain; however, these regions were not predicted as the EGF-like domain using the CDS program. Because Cadherin repeat and EGF-like domain are defined in a low-stringent manner, it is ambiguous at present whether regions distantly related to Cadherin repeat and EGF-like domain are functional or not. Domain architectures of human FAT1, FAT2 FAT3, FAT4 and *Drosophila* Fat and Fat-like (Fatl) were illustrated based on the results of the CDS program using each RefSeq as a query sequence. Domain-architecture topologies of the region between Cadherin repeats and the transmembrane domain of human FAT1, FAT2, FAT3 and *Drosophila* Fatl are a Laminin-G-like domain followed by multiple EGF-like domains, whereas those of human FAT4 and *Drosophila* Fat are multiple EGF-like domains followed by two Laminin-G-like domains (Fig. 2). Phylogenetic analyses on human and *Drosophila* FAT family proteins revealed that only FAT4 is located within the same branch as *Drosophila* Fat (Fig. 2). Together, these facts indicate that human FAT1, FAT2 and FAT3 are orthologs of *Drosophila* Fatl, and that human FAT4 is the ortholog of *Drosophila* Fat.

## 3. Processing of FAT proteins

FAT1 and FAT4 undergo the first proteolytic cleavage in the extracellular region by Furin during their maturation step, which gives rise to non-covalent heteodimer consisting of a larger subunit corresponding to the most part of the extracellular region and a smaller subunit containing the transmembrane and cytoplasmic regions (22,32). Artificial FAT proteins undergo the second proteolytic cleavage by γ-secretase and the release of intracellular region, which is similar to the ligand-dependent processing of NOTCH receptors (33). However, evidence of the ligand-dependent second cleavage of endogenous FAT proteins remains unclear.

## 4. Signaling and function of FAT1 and FAT4

**Figure 3:**
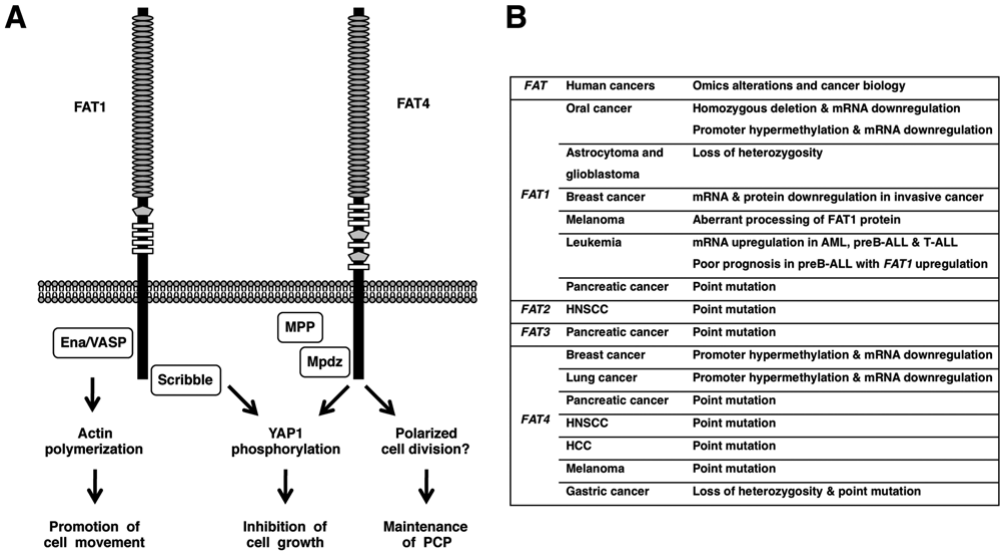
Function and cancer genomics of FATs. (A) FAT1 and FAT4 signaling cascades. FAT1 interacts with Ena/VASP to induce actin polymerization at lamellipodia and filopodia, which is involved in the promotion of cell movement. FAT1 interacts with Scribble to induce YAP1 phosphorylation, which is involved in the inhibition of cell growth. FAT4 interacts with MPDZ (MUPP1) to recruit membrane-associated guanylate kinase MPP5 (PALS1). FAT4 is involved in the maintenance of PCP as well as the inhibition of cell proliferation. (B) Omics alterations of *FAT* family members in human cancers. AML, acute myeloid leukemia; ALL, acute lymphoblastic leukemia; HNSCC, head and neck squamous cell carcinoma; HCC, hepatocellular carcinoma.

Dachsous1 (DCHS1) and Dachsous2 (DCHS2) are mammalian orthologs of *Drosophila* Dachsous (Fig. 1B); however, heterophilic interaction between extracellular regions of FAT1 and Dachsous1/2 remains unknown. On the other hand, intracellular region of FAT1 directly interacts with Ena/VASP, HOMER, KIF5C and Scribble proteins (34–37). Ena/VASP and HOMER are EVH1-domain proteins binding to the cytoplasmic FPPPPEDF motif of Fat1 in a mutually competitive manner. Because Ena/VASP proteins inhibit actin capping and induce actin polymerization, Fat1-mediated recruitment of Ena/VAPS proteins to the leading edge of lamellipodia and the tip of filopodia results in the promotion of cell migration (34,35). Scribble proteins are scaffold proteins with multiple PDZ domains binding to the C-terminal HTEV motif of Fat1. Fat1 and Scribble are synergistically involved in the suppression of cystogenesis phenotype through the inhibition of Yap1 signaling (37). Fat1 knockdown in vascular smooth muscle cells results in decreased migration and enhanced proliferation (38). FAT1 is involved in promotion of actin-mediated cell migration as well as inhibition of YAP1-mediated cell proliferation (Fig. 3A).

Fat4 heterophilically interacts with Dachsous1 at the apical portion of cell-cell boundaries of neural progenitor cells, where intracellular region of Fat4 directly interacts with Mpdz/Mupp1-Mpp5/Pals1 complex (39). Mpp1, Mpp2, Mpp3, Mpp4, Mpp5, Mpp6/Pals2 and Mpp7 are membrane-associated guanylate kinase (MAGUK) homologs of *Drosophila* Stardust (Sdt), which is involved in the maintenance of apicobasal polarity in epithelial tissues (40). *Fat4* knockout mice die at birth, which are manifested by stereocilia disorientation in the inner ear, loop tail, broader neural tube and renal cysts (41). Disorientation of cochlear hair cells is the typical phenotype of the mammalian PCP defect in *Vangl2*, *Celsr1* or *Dvl1/Dvl2* mutant mice (42). Loop tail and neural tube abnormalities are also observed in *Vangl2* mutant mice (43) and renal cystogenesis is synergistically enhanced in *Fat4*^−/−^*Vangl2*^−/+^ mice (42). *Fat4* knockdown in neural tube results in an increase of a subset of neural progenitors and differentiated Lim1^+^/Lim2^+^ neurons via downregulation of Yap1 phosphorylation (44). FAT4 is involved in the maintenance of PCP as well as inhibition of YAP1-mediated cell proliferation (Fig. 3A).

## 5. Cancer genomics of *FAT* family genes

The human *FAT1* gene is homozygously deleted in 23% of oral cancer cell lines and in 80% of primary oral cancer cases and *FAT1* mRNA expression is repressed in oral cancer cell lines due to homozygous deletion and/or promoter CpG hypermethylation (45). Loss of heterozygosity (LOH) of the *FAT1* gene occurs in 42% of low grade diffuse astrocytoma and 63% of glioblastoma multiforme (46). *FAT1* mRNA level in ductal carcinoma *in situ* is significantly higher than that in invasive breast cancer and *FAT1* knockdown promotes progression from ductal carcinoma *in situ* to invasive breast cancer (47). *FAT1* mRNA expression is upregulated in 11% of acute myeloid leukemia (AML), 29% of preB acute lymphoblastic leukemia (ALL) and 63% of T-ALL, and *FAT1* upregulation in preB-ALL is associated with shorter relapse-free survival as well as shorter overall survival (48). FAT1 immunoreactivity is strong in 29% of cholangiocarcinoma (49).

The mouse *Fat3* mRNA is significantly downregulated in lung adenocarcinoma occurred in transgenic mice expressing wild-type *Raf1* transgene under the control of the human *SP*-*C* (surfactant protein C) promoter (50).

The mouse *Fat4* gene is inactivated owing to LOH and promoter CpG hypermethylation in subcutaneous tumor induced by Cre/LoxP-mediated random chromosomal deletion (51). Tumor growth is inhibited by re-introduction of *Fat4* gene into cells derived from the cutaneous tumor. Relative YAP1 activity is significantly upregulated as a result of *Fat4* repression.

The human *FAT4* mRNA expression is repressed in 3 out of 6 breast cancer cell lines and in 3 out of 5 cases of primary breast cancers, partially due to promoter CpG hypermethylation (51). *FAT4* promoter is hypermethylated in 7 out of 18 cases of lung adenocarcinoma (stage I) and *FAT4* mRNA is downregulated in 18 out of 23 cases of non-small cell lung tumors (stage I or II) (52).

Using the whole-exome sequencing approach, non-synonymous mutations of human *FAT1*, *FAT3* and *FAT4* genes are detected in 1 each, and 2 out of 24 pancreatic cancer samples, respectively (53). Non-synonymous mutations of human *FAT2* and *FAT4* genes are detected in 1 and 2 out of 32 cases of head and neck squamous cell carcinoma (HNSCC), respectively (54). Non-synonymous *FAT4* mutation is detected in 1 out of 10 cases of hepatocellular carcinoma using the whole-exome sequencing approach (55). Non-synonymous *FAT4* mutations are also detected in 4 out of 6 cases of melanomas using the whole-exome sequencing approach and in 2 out of additional 9 cases of melanomas using the candidate-exons sequencing approach (56). Non-synonymous *FAT4* mutations are detected in 2 out of 15 cases of gastric cancers using the whole-exome approach and in 4 out of additional 95 cases of gastric cancers using the candidate-exon approach (57). Among the human *FAT* gene family, *FAT4* gene is recurrently mutated in several types of human cancers, such as melanoma (40%), pancreatic cancer (8%), HNSCC (6%) and gastric cancer (5%).

## 6. Conclusion

*FAT1* is downregulated in oral cancer and invasive breast cancer due to deletion and/or epigenetic silencing, whereas *FAT1* is upregulated in leukemia and prognosis of preB-ALL with *FAT1* upregulation is poor. *FAT4* is mutated in several types of human cancer, such as melanoma, pancreatic cancer and gastric cancer (Fig. 3B). FAT1 and FAT4 suppress tumor growth through Hippo signaling activation, while FAT1 promotes tumor migration through actin polymerization at lamellipodia and filopodia. Together, these facts indicate that *FAT1* is tumor suppressive or oncogenic in a context-dependent manner and that *FAT4* is preferentially tumor suppressive.

## 7. Perspectives

*Drosophila* Fat is involved in the tumor suppression via phosphorylation-mediated functional inhibition of Yorkie through indirect activation of the Expanded-Hippo-Warts signaling cascade (Fig. 1A). Expanded interacts with Merlin and Kibra to activate the Hippo signaling cascade, while Salvador and Mats are involved in the regulation of Hippo and Warts kinases, respectively (Fig. 1A). FRMD1 and FRMD6 are human orthologs of *Drosophila* Expanded; NF2 is the human ortholog of *Drosophila* Merlin; WWC1 and WWC2 are human orthologs of *Drosophila* Kibra; SAV1 is the human ortholog of *Drosophila* Salvador; STK3 and STK4 are human orthologs of *Drosophila* Hippo; MOB1A and MOB1B are human orthologs of *Drosophila* Mats; LATS1 and LATS2 are human orthologs of *Drosophila* Warts; YAP1 and WWTR1 (TAZ) are human orthologs of *Drosophila* Yorkie (Fig. 1B). Copy number aberration, translocation and point mutation of human *FAT1, FAT2, FAT3, FAT4, FRMD1, FRMD6, NF2, WWC1, WWC2, SAV1, STK3, STK4, MOB1A, MOB1B, LATS1, LATS2, YAP1* and *WWTR1* genes should be comprehensively investigated in various types of human cancers using high-throughput sequencing technology to elucidate the mutation landscape of the FAT-Hippo signaling cascades.

YAP1 and WWTR1 directly interact with β-catenin and Hippo signaling-induced phosphorylation of YAP1 results in the inhibition of the canonical WNT signaling cascade (58). WNT signaling cascades crosstalk with FGF, Notch, Hedgehog and TGFβ/BMP signaling cascades to constitute the stem-cell signaling network (59). Because Hippo-YAP1/WWTR1 signaling cascade is located at the crossroads of adhesion signaling, G-protein-coupled receptor (GPCR) signaling, receptor tyrosine kinase (RTK) signaling and stem cell biology (12,60–62), cancer genomics of the FAT signaling cascades could be applied for diagnostics, prognostics and therapeutics in the era of personalized medicine.

## References

[1] Bryant PJ, Huettner B, Held LI, Ryerse J, Szidonya J (1988). Mutations at the *fat* locus interfere with cell proliferation control and epithelial morphogenesis in *Drosophila*. Dev Biol.

[2] Woods DF, Bryant PJ (1991). The *discs-large* tumor suppressor gene of *Drosophila* encodes a guanylate kinase homolog localized at septate junctions. Cell.

[3] Strand D, Raska I, Mechler BM (1994). The *Drosophila lethal(2) giant larvae* tumor suppressor protein is a component of the cytoskeleton. J Cell Biol.

[4] Justice RW, Zilian O, Woods DF, Noll M, Bryant PJ (1995). The *Drosophila* tumor suppressor gene *warts* encodes a homolog of human myotonic dystrophy kinase and is required for the control of cell shape and proliferation. Genes Dev.

[5] Bilder D, Perrimon N (2000). Localization of apical epithelial determinants by the basolateral PDZ protein Scribble. Nature.

[6] Tapon N, Harvey KF, Bell DW (2002). *salvador* promotes both cell cycle exit and apoptosis in *Drosophila* and is mutated in human cancer cell lines. Cell.

[7] Pantalacci S, Tapon N, Léopold P (2003). The Salvador partner Hippo promotes apoptosis and cell-cycle exit in *Drosophila*. Nat Cell Biol.

[8] Bryant PJ, Watson KL, Justice RW, Woods DF (1993). Tumor suppressor genes encoding proteins required for cell interactions and signal transduction in *Drosophila*. Dev Suppl.

[9] Hariharan IK, Bilder D (2006). Regulation of imaginal disc growth by tumor-suppressor genes in *Drosophila*. Annu Rev Genet.

[10] Saucedo LJ, Edgar BA (2007). Filling out the Hippo pathway. Nat Rev Mol Cell Biol.

[11] Reddy BV, Irvine KD (2008). The Fat and Warts signaling pathways: new insights into their regulation, mechanism and conservation. Development.

[12] Pan D (2010). The hippo signaling pathway in development and cancer. Dev Cell.

[13] Genevet A, Tapon N (2011). The Hippo pathway and apico-basal cell polarity. Biochem J.

[14] Yang CH, Axelrod JD, Simon MA (2002). Regulation of Frizzled by fat-like cadherins during planar polarity signaling in the *Drosophila* compound eye. Cell.

[15] Strutt H, Strutt D (2002). Nonautonomous planar polarity patterning in *Drosophila: dishevelled*-independent functions of *frizzled*. Dev Cell.

[16] Axelrod JD (2009). Progress and challenges in understanding planar cell polarity signaling. Semin Cell Dev Biol.

[17] Djiane A, Mlodzik M (2010). The *Drosophila* GIPC homologue can modulate myosin based processes and planar cell polarity but is not essential for development. PLoS One.

[18] Axelrod JD, Miller JR, Shulman JM, Moon RT, Perrimon N (1998). Differential recruitment of Dishevelled provides signaling specificity in the planar cell polarity and Wingless signaling pathways. Genes Dev.

[19] Mlodzik M (2002). Planar cell polarization: do the same mechanisms regulate *Drosophila* tissue polarity and vertebrate gastrulation?. Trends Genet.

[20] Katoh M (2005). WNT/PCP signaling pathway and human cancer (Review). Oncol Rep.

[21] Ishikawa HO, Takeuchi H, Haltiwanger RS, Irvine KD (2008). Four-jointed is a Golgi kinase that phosphorylates a subset of cadherin domains. Science.

[22] Sopko R, McNeill H (2009). The skinny on Fat: an enormous cadherin that regulates cell adhesion, tissue growth, and planar cell polarity. Curr Opin Cell Biol.

[23] Thomas C, Strutt D (2012). The roles of the cadherins Fat and Dachsous in planar polarity specification in *Drosophila*. Dev Dyn.

[24] Bosveld F, Bonnet I, Guirao B (2012). Mechanical control of morphogenesis by Fat/Dachsous/Four-jointed planar cell polarity pathway. Science.

[25] Mahoney PA, Weber U, Onofrechuk P, Biessmann H, Bryant PJ, Goodman CS (1991). The *fat* tumor suppressor gene in *Drosophila* encodes a novel member of the cadherin gene superfamily. Cell.

[26] Tanoue T, Takeichi M (2005). New insights into Fat cadherins. J Cell Sci.

[27] Hulpiau P, van Roy F (2009). Molecular evolution of the cadherin superfamily. Int J Biochem Cell Biol.

[28] Dunne J, Hanby AM, Poulsom R (1995). Molecular cloning and tissue expression of *FAT*, the human homologue of the *Drosophila fat* gene that is located on chromosome 4q34-q35 and encodes a putative adhesion molecule. Genomics.

[29] Wu Q, Maniatis T (2000). Large exons encoding multiple ectodomains are a characteristic feature of protocadherin genes. Proc Natl Acad Sci USA.

[30] Höng JC, Ivanov NV, Hodor P (2004). Identification of new human cadherin genes using a combination of protein motif search and gene finding methods. J Mol Biol.

[31] Katoh Y, Katoh M (2006). Comparative integromics on *FAT1, FAT2, FAT3* and *FAT4*. Int J Mol Med.

[32] Sadeqzadeh E, de Bock CE, Zhang XD (2011). Dual processing of FAT1 cadherin protein by human melanoma cells generates distinct protein products. J Biol Chem.

[33] Magg T, Schreiner D, Solis GP, Bade EG, Hofer HW (2005). Processing of the human protocadherin Fat1 and translocation of its cytoplasmic domain to the nucleus. Exp Cell Res.

[34] Tanoue T, Takeichi M (2004). Mammalian Fat1 cadherin regulates actin dynamics and cell-cell contact. J Cell Biol.

[35] Moeller MJ, Soofi A, Braun GS (2004). Protocadherin FAT1 binds Ena/VASP proteins and is necessary for actin dynamics and cell polarization. EMBO J.

[36] Schreiner D, Müller K, Hofer HW (2006). The intracellular domain of the human protocadherin hFat1 interacts with Homer signalling scaffolding proteins. FEBS Lett.

[37] Skouloudaki K, Puetz M, Simons M (2009). Scribble participates in Hippo signaling and is required for normal zebrafish pronephros development. Proc Natl Acad Sci USA.

[38] Hou R, Liu L, Anees S, Hiroyasu S, Sibinga NE (2006). The Fat1 cadherin integrates vascular smooth muscle cell growth and migration signals. J Cell Biol.

[39] Ishiuchi T, Misaki K, Yonemura S, Takeichi M, Tanoue T (2009). Mammalian Fat and Dachsous cadherins regulate apical membrane organization in the embryonic cerebral cortex. J Cell Biol.

[40] Katoh M, Katoh M (2004). Identification and characterization of human *MPP7* gene and mouse *Mpp7* gene *in silico*. Int J Mol Med.

[41] Saburi S, Hester I, Fischer E (2008). Loss of *Fat4* disrupts PCP signaling and oriented cell division and leads to cystic kidney disease. Nat Genet.

[42] Jones C, Chen P (2007). Planar cell polarity signaling in vertebrates. Bioessays.

[43] Kibar Z, Vogan KJ, Groulx N, Justice MJ, Underhill DA, Gros P (2001). *Ltap*, a mammalian homolog of *Drosophila Strabismus/Van Gogh*, is altered in the mouse neural tube mutant Loop-tail. Nat Genet.

[44] van Hateren NJ, Das RM, Hautbergue GM, Borycki AG, Placzek M, Wilson SA (2011). FatJ acts via the Hippo mediator Yap1 to restrict the size of neural progenitor cell pools. Development.

[45] Nakaya K, Yamagata HD, Arita N (2007). Identification of homozygous deletions of tumor suppressor gene *FAT* in oral cancer using CGH-array. Oncogene.

[46] Chosdol K, Misra A, Puri S (2009). Frequent loss of heterozygosity and altered expression of the candidate tumor suppressor gene *‘FAT’* in human astrocytic tumors. BMC Cancer.

[47] Lee S, Stewart S, Nagtegaal I (2012). Differentially expressed genes regulating the progression of ductal carcinoma in situ to invasive breast cancer. Cancer Res.

[48] de Bock CE, Ardjmand A, Molloy TJ (2012). The Fat1 cadherin is overexpressed and an independent prognostic factor for survival in paired diagnosis-relapse samples of precursor B-cell acute lymphoblastic leukemia. Leukemia.

[49] Settakorn J, Kaewpila N, Burns GF, Leong AS (2005). FAT, E-cadherin, β-catenin, HER 2/neu, Ki67 immuno-expression, and histological grade in intrahepatic cholangiocarcinoma. J Clin Pathol.

[50] Rohrbeck A, Borlak J (2009). Cancer genomics identifies regulatory gene networks associated with the transition from dysplasia to advanced lung adenocarcinomas induced by c-Raf-1. PLoS One.

[51] Qi C, Zhu YT, Hu L, Zhu YJ (2009). Identification of *Fat4* as a candidate tumor suppressor gene in breast cancers. Int J Cancer.

[52] Rauch TA, Wang Z, Wu X, Kernstine KH, Riggs AD, Pfeifer GP (2012). DNA methylation biomarkers for lung cancer. Tumour Biol.

[53] Jones S, Zhang X, Parsons DW (2008). Core signaling pathways in human pancreatic cancers revealed by global genomic analyses. Science.

[54] Agrawal N, Frederick MJ, Pickering CR (2011). Exome sequencing of head and neck squamous cell carcinoma reveals inactivating mutations in *NOTCH1*. Science.

[55] Li M, Zhao H, Zhang X (2011). Inactivating mutations of the chromatin remodeling gene *ARID2* in hepatocellular carcinoma. Nat Genet.

[56] Nikolaev SI, Rimoldi D, Iseli C (2011). Exome sequencing identifies recurrent somatic *MAP2K1* and *MAP2K2* mutations in melanoma. Nat Genet.

[57] Zang ZJ, Cutcutache I, Poon SL (2012). Exome sequencing of gastric adenocarcinoma identifies recurrent somatic mutations in cell adhesion and chromatin remodeling genes. Nat Genet.

[58] Imajo M, Miyatake K, Iimura A, Miyamoto A, Nishida E (2012). A molecular mechanism that links Hippo signalling to the inhibition of Wnt/β-catenin signalling. EMBO J.

[59] Katoh M, Katoh M (2007). WNT signaling pathway and stem cell signaling network. Clin Cancer Res.

[60] Yu FX, Zhao B, Panupinthu N (2012). Regulation of the Hippo-YAP pathway by G-Protein-coupled receptor signaling. Cell.

[61] Huang W, Lv X, Liu C (2012). The N-terminal phosphodegron targets TAZ/WWTR1 protein for SCF β-TrCP-dependent degradation in response to phosphatidylinositol 3-kinase inhibition. J Biol Chem.

[62] Cordenonsi M, Zanconato F, Azzolin L (2011). The Hippo transducer TAZ confers cancer stem cell-related traits on breast cancer cells. Cell.

